# Analysis of imidacloprid residues in mango, cowpea and water samples based on portable molecular imprinting sensors

**DOI:** 10.1371/journal.pone.0257042

**Published:** 2021-09-02

**Authors:** Sihua Peng, Shuyan Yang, Xi Zhang, Jingjing Jia, Qiulin Chen, Yuyang Lian, Aqiang Wang, Bei Zeng, Heming Yang, Jinlei Li, Jianguo Dan, Jianjun Liao, Shihao Zhou

**Affiliations:** 1 College of Plant Protection, Hainan University, Hainan, Haikou, China; 2 Key Laboratory of Germplasm Resources Biology of Tropical Special Ornamental Plants of Hainan Province, College of Forestry, Hainan University, Haikou, China; 3 Institute of Plant Protection, Hainan Academy of Agricultural Sciences, Hainan, Haikou, China; 4 College of Ecology and Environment, Hainan University, Hainan, Haikou, China; Chinese Academy of Agricultural Sciences Institute of Plant Protection, CHINA

## Abstract

Imidacloprid is a neonicotinoid insecticide widely used in the production and cultivation of crops. In recent years, the extensive use of imidacloprid in agricultural production has resulted in large amounts of pesticide residues in agricultural products and the environment. Therefore, it is necessary to establish a rapid, accurate, sensitive and convenient method for detecting imidacloprid pesticide residues to ensure the safety of agricultural products and the environment. To clarify how to use the molecular imprinting method for the electrochemical rapid residue detection of imidacloprid. This paper selected reduced graphene oxide and gold nanoparticles as modifiers modified on screen-printed carbon electrodes (SPCE) chitosan as a functional monomer, and imidacloprid as template molecule to prepare molecularly imprinted polymer, and applied this sensor to the residue detection of imidacloprid. The results showed that the concentration of imidacloprid showed a good linear relationship with the peak response current, and the detection limit of imidacloprid was 0.5 μM, while the sensor had good repeatability and interference resistance. The recoveries of imidacloprid spiked on three samples, mango, cowpea and water, were in the range of 90–110% (relative standard deviation, RSD<5%), which proved the practicality and feasibility of the assay established in this paper. The results of this paper can be used as a basis for the research on the detection of imidacloprid pesticide residues in food or environment.

## Introduction

Imidacloprid is a largely commercialized first generation neonicotinoid insecticide Imidacloprid [1], which has gastric, thixotropic, and systemic activities, as well as high efficiency, low toxicity, and broad-spectrum characteristics [2]. It is widely used to control thrips and mealybugs during mango and cowpea cultivation [3,4]. However, relevant studies have shown that the use of pesticides will affect non-target organisms such as *Trichogramma*, *Harmonia axyridis*, and honeybees [5–7]. In addition, imidacloprid can affect the heart, liver and other organs of mammals, and stimulate the gastrointestinal tract, affect the nervous and reproductive systems and even die [8–10]. In recent years, the widespread use of imidacloprid in agricultural production has resulted in huge amounts of pesticide residues in food and the environment, posing health risks and polluting the ecosystem [11–13]. At the same time, some experiments have confirmed that it is difficult to wash vegetables and fruits with water or detergent to clean the excessive pesticide residues below toxic levels [14,15].

Currently, the main methods for the detection of imidacloprid are gas chromatography (GC) [16,17], gas chromatography/mass spectrometry (GC-MS) [18–20], high performance liquid chromatography (HPLC) [21–23], liquid chromatography/mass spectrometry (LC-MS) [24,25], enzyme immunoassay [26,27], and capillary electrophoresis [28]. Although the above methods retain their sensitivity and accuracy, they require a lot of instrumentation, and the operation procedures are inconvenient and time-consuming, as well as the need for skilled operating techniques. As a result, pesticide residues cannot be determined quickly, so there is a need to develop more portable and accurate new energy to residue detection tools. Electrochemical detection has greater advantages in pesticide residue detection due to its good stability, miniaturization, high sensitivity and rapid response [29–31].

Molecular imprinting technique (MIT) crosslinks functional monomers and template molecules through cross linking agents to generate stable polymers, and eluents elute the target molecules to produce molecularly imprinted polymers (MIP) [32,33]. Molecular imprinting techniques are currently used to detect drug residues in food, environmental monitoring, etc. In the detection of pesticide residues, molecular imprinting TiO2 photocatalyst has been synthesized by sol-gel technology. It has been tested whether the pollutants contain 2,4D herbicide and imidacloprid [34], and the disposable Molecularly Imprinted Electrochemical sensor for the detection of organophosphorus insecticide monophosphate (PAS) [35]. Combining molecular imprinting technique with electrochemical detection technique combines the advantages of both detection techniques [36].

The screen-printed carbon electrode (SPCE) used in this study has the advantages of small size, low cost, disposability, simplicity and portability compared to the conventional glassy carbon electrode, and ease of use with portable detection devices. Graphene oxide (GO) has good electron transfer properties, and reduced Graphene Oxide (rGO) was obtained by electrochemical reduction of GO [37–39]. Electrodeposition of nanogold on the surface of screen-printed electrodes greatly increased the conductivity of the electrodes [40]. Chitosan (CS) can produce powerful adsorption of metal ions, halogen ions and many organic substances [41–44]. Using the CS structure that can bind functional groups, molecularly imprinted electrochemical sensors with good interference resistance can be fabricated [45].

In this study, a portable molecularly imprinted sensor (IMI-MIP/Au/rGO/GCE) of imidacloprid was prepared by electroreducing graphene oxide on screen-printed electrodes, modifying gold nanoparticles. The chitosan was used as a functional matrix, imidacloprid as a template molecule, and glutaraldehyde as a cross linking agent under specific conditions, and subsequently analyzing the electrochemical behavior of the prepared electrochemical sensor. This method was applied to the rapid analysis of imidacloprid in water samples, mango, and cowpea samples.

## Materials and methods

### Materials and reagents

Chitosan, graphene oxide, sodium dihydrogen phosphate, disodium hydrogen phosphate, glutaraldehyde and imidacloprid (analytical purity) were purchased from Shanghai Aladdin Bio-Chem Technology Co. Ltd. The potassium hexacyanoferric(II) acid trihydrate and acetic acid (analytical purity) were purchased from Xilong Scientific Co. Ltd. (Chemical Reagent Factory). Acetonitrile (chromatographic pure) was purchased from Shanghai Macklin Biochemical Co. The mangoes and cowpeas were purchased from the fruit shop and farmers’ market at the east gate of Danzhou Campus of Hainan University, Danzhou City, China. The water samples were collected from the river beside the Botanical Garden in Danzhou City (N19°30′43″, E109°30′1″).

### Instruments and equipment

The electrochemical workstation (CHI660E) was purchased from Shanghai Chenhua Instruments Co. Ltd, electronic analytical balance (Sartorius Sartorius), tabletop high-speed frozen centrifuge H3-18KR (Hangzhou Gengyu Instruments Co. Ltd.), precision type water purifier FST-111-TH100 (Puliflex), screen-printed carbon electrode E100.

### Preparation of pesticide standard solution

Accurately weigh 1000 mol of imidacloprid in a 100 ml volumetric flask, prepared the standard pesticide solution of 10000 μmol/L with PBS solvent, and stored it in a room-temperature environment. The PBS was used to further dilute the 10,000 μmol/L pesticide solution into a 0.5, 1.0, 1.5, 2.0, 2.5, and 3.0 μmol/L series of standard pesticide solutions.

### Sample pretreatment

The mangoes were properly cleaned, dried, cored, and sliced. Weighted 10g of homogenate in a 50mL centrifuge tube, added 10mL of 1% acetic acid acetonitrile solution, vortex for 3 minutes, then centrifuged at 8000r/min for 5 minutes. The supernatant was filtered and utilized in the experiment. The sample solutions containing 1, 2, and 3 μmol/L imidacloprid were prepared by adding the corresponding amount of imidacloprid to the supernatant. Cowpea samples were prepared as above. The river water was filtered and added to the corresponding amount of imidacloprid to prepare the sample solution containing 1, 2 and 3 μmol/L imidacloprid.

### Preparation of modifiers

Imidacloprid chitosan stock solution: Mixed 2g of chitosan with 2% glacial acetic acid and fixed the volume to 100mL, mixed with ultrasonic until the chitosan is completely dissolved, left it overnight, and then added the quantitative imidacloprid prodrug to make 1.0 mmol/L.

HAuCl_4_ stock solution: Used 0.5mol/LH_2_SO_4_ as a solvent and added quantitative HAuCl_4_ to configure the deposition solution containing 0.2% HAuCl_4_.

### Molecular imprinting sensor preparation

Referring to Bo Liang *et al*. [46], GO was selected as a modifier to increase the conductivity of the electrode. The the screen-printed carbon electrode was cleaned and dried with water and set aside, 80 μL of 2 mg/mL GO solution was added dropwise on the electrode surface, and cyclic voltammetry (CV) was used to scan 16 cycles at 0.05 V/S in the potential interval of -1.7–0.2 V to rinse excess GO solution and dried. 80 uL of HAuCl_4_ deposition solution was applied dropwise to the electrode and deposited at a constant potential of -0.25 V for 180 s. After completion, the electrode was removed, rinsed and dried.

A certain amount of imidacloprid chitosan stock solution was added dropwise to the electrode to cover the electrode and deposited at the constant potential at -1.0 V for 300 s to polymerize both imidacloprid and chitosan electrode surface [47]. After rinsing and drying, 4μL of 0.1% glutaraldehyde solution was added dropwise to the electrode. The template molecules were eluted off using cyclic voltammetry scanning for 50 revolutions in the potential range of -0.4–0.8 V. After completion; the electrode was removed and washed.

### Electrochemical characterization

The electrode/sensor was immersed in a 0.1 mol/L KCl solution containing 5.0 mmol/L K_3_Fe(CN_6_), and cyclic voltammetry (CV) scan was performed for 5 turns in the potential range of -0.2–0.6 V to obtain the voltammogram of the electrode/sensor.

### Sensor performance DPV test

The prepared sensor was scanned by differential pulse voltammetry (DPV) with a homemade portable sensor in a 0.1 mol/L KCl solution containing 5.0 mmol/L K_3_Fe(CN_6_). The peak current of the sensor at this time was recorded as I_0_; after that, the above sensor was immersed into 0.01 mol/L PBS containing different concentrations of imidacloprid solution with pH = 7 After the inhibition was completed, the sensor was scanned by differential pulse voltammetry (DPV), and the peak current of sensor was recorded as I. The inhibition rate (I%) of the sensor was calculated according to the following formula.


$$I\%=\frac{I_{0}-I}{I_{0}}\times 100\%$$

### Repeatability test

The same sensor was immersed in PBS solution containing 2μmol/L imidacloprid for 30min, and 80μL of 0.1mol/L KCl solution containing 5.0mmol/L K_3_Fe(CN_6_) was dropped and scanned by differential pulse voltammetry, and the peak current values were recorded. This operation was performed for 7 consecutive days, and the relative standard deviation (RSD) was calculated.

### Interference resistance test

Added 5, 10, and 20 μmol/L thiamethoxam solution to 1μmol/L imidacloprid solution and mixed well. This mixture was used as the interference solution. The sensor was immersed in the above solution for 30 min according to the gradient. Then 80 μL of 0.1 mol/L KCl solution containing 5.0 mmol/L K3Fe(CN6) was dropped and scanned by differential pulse voltammetry. The peak current values were recorded to calculate the current values of both before and after the addition of thiamethoxam solution and their current differences.

### Sample recovery test

After the sensor was immersed in the sample containing different concentrations of imidacloprid and dried for 30 min, 80 μL of 0.1 mol/L KCl solution containing 5.0 mmol/L K_3_Fe(CN_6_) was dropped, scanned by differential pulse voltammetry, and the peak current values were recorded. The recoveries and relative standard deviations (RSDs) were calculated.

## Results

### Electrochemical characterization analysis results

**Fig 1:**
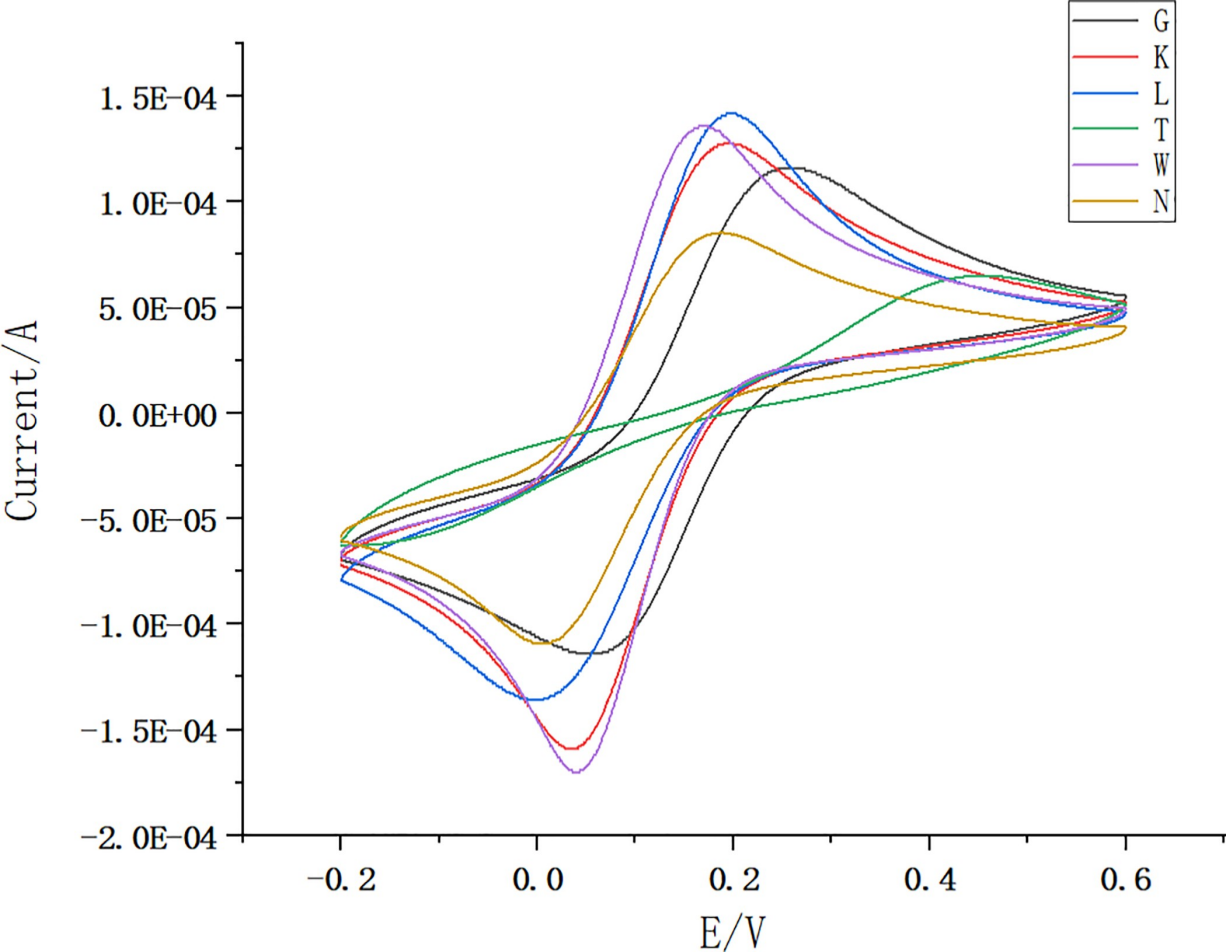
Cyclic voltammetric scan curves on different electrodes and sensors. Bare electrode CV (bare SPCE; purple; A), GO post-electrode (rGO/SPCE; navy blue; B), Electrodeposited HAuCl4 solution post-electrode (Au/rGO/SPCE; wathet; C), Polymerized IMI molecular polymer post-electrode (green; D), Eluted post-electrode (IMI-MIP/Au/rGO/SPCE; orange; E), Non-imprinted sensor eluted post-electrode (CK) (N-IMI-MIP/Au/rGO/SPCE; red; F).

Cyclic voltammetry analysis was performed for each electrode separately, and the results are shown in Fig 1. The results show that the peak current of the electrode after deposition of chloroauric acid is the highest, indicating that chloroauric acid successfully modifies the surface of the upper electrode, increasing the conductivity of the electrode and increasing the peak current at the same time. The current after the elution of the imprinted sensor was larger than that after the elution of the non-imprinted sensor, indicating that the imprinted sites on the surface of the imprinted film were very good for the imidacloprid molecule. Meanwhile, the preparation process of the non-imprinted sensor does not involve the imprinted molecules. It cannot cross-link the growing polymer chains, resulting in the thickening of the polymeric film and narrowing the intermolecular gap, and thus its peak current is small.

### Sensor performance test results

**Fig 2:**
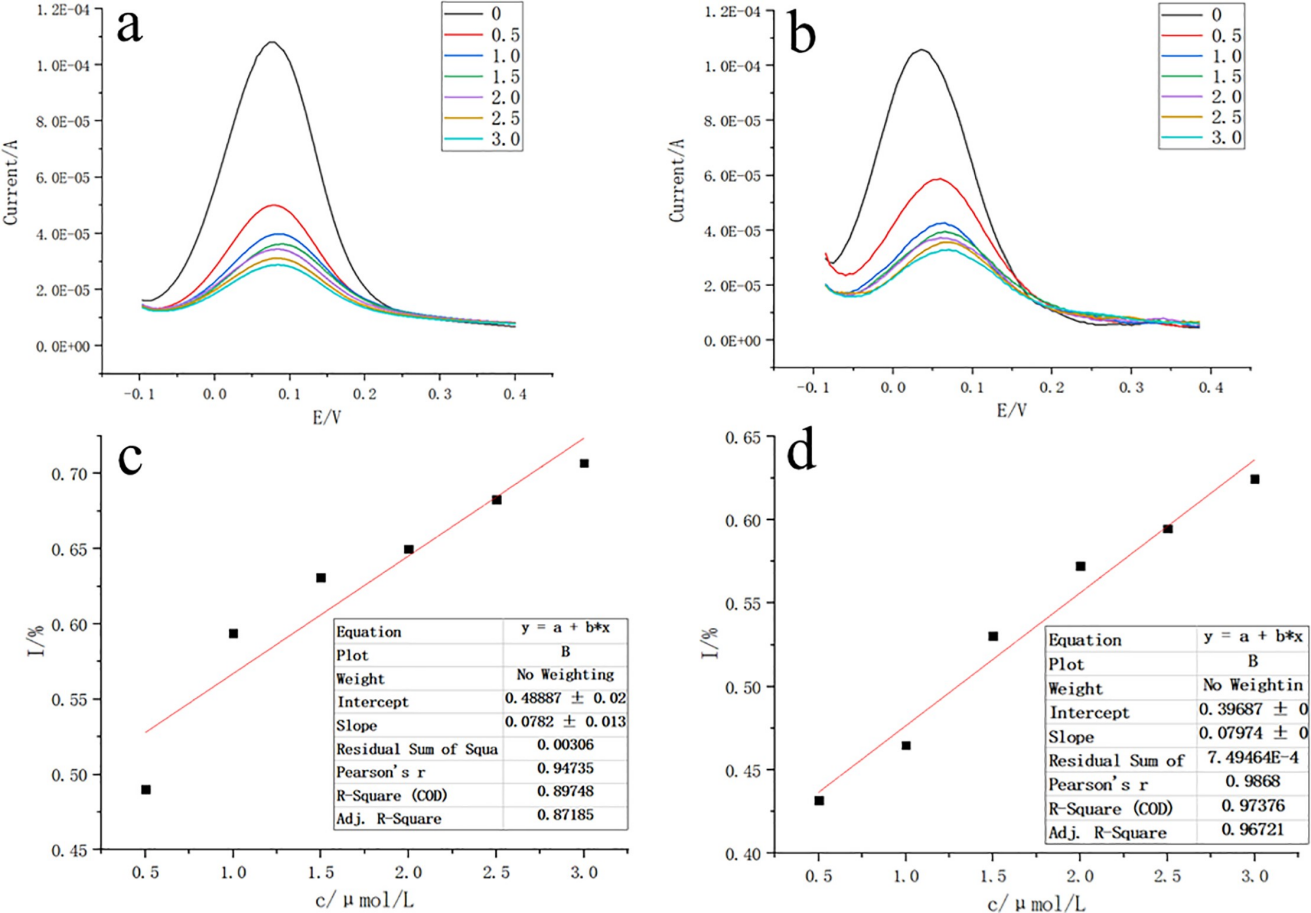
Comparison of electrochemical workstation and portable sensors. (a) DPV curves of portable sensor based on cell phone for different concentrations of Imidacloprid. (b) DPV curves of electrochemical workstation for different concentrations of Imidacloprid. (c) Inhibition rate curves of portable sensor based on cell phone for different concentrations of Imidacloprid. (d) Inhibition rate curves of electrochemical workstation for different concentrations of Imidacloprid. Note: 0, 0.5, 1.0, 1.5, 2.0, 2.5, 3.0 are different concentrations of Imidacloprid, where the concentration units are μmol/L.

The results of the differential pulse analysis of the prepared molecularly imprinted electrochemical sensor on the electrochemical workstation and the mobile phone-based portable sensor are shown in Fig 2. The response currents of both sensors changed with the gradient of imidacloprid concentration, and the corresponding response currents decreased when the imidacloprid concentration increased. Therefore, there is a good linear relationship between the response current and the pesticide concentration within a certain concentration range. The comparison between the two shows that the linear fit of the detection results of the mobile phone-based portable sensor is higher than that of the electrochemical workstation detection results.

### Results of repeatability test

Testing was done using the repeatability test technique. The peak current and RSD values for seven days are given in Table 1. The RSD value for 7 consecutive days was 4.16%, and the peak current did not decrease significantly after 7 days, indicating that the prepared sensor has good repeatability.

**Table 1:** Repeatability test results.

Time(day)	Current/A	RSD
1	5.46E-05	4.16%
2	5.09E-05	
3	5.32E-05	
4	5.56E-05	
5	5.57E-05	
6	5.55E-05	
7	5.05E-05	

### Results of anti-disturbance test

The 1μmol/L imidacloprid (IMI) and imidacloprid solutions containing 5μmol/L, 10μmol/L and 20μmol/L thiamethoxam (TMX) were tested using the method of interference resistance test (Table 2). The 1μmol/L imidacloprid and 1μmol/L imidacloprid mixed 5μmol/L, 10μmol/L and 20μmol/L thiamethoxam had similar peak currents. The differences in their inhibition rates were 1.90%, 3.70% and 4.70%, which were less than 5%, respectively. The above results indicate that this molecularly imprinted sensor has good interference resistance.

**Table 2:** Anti-interference test results.

Sample	1μmol/L IMI	1μmol/L IMI+5μmol/L TMX	1μmol/L IMI+10μmol/L TMX	1μmol/L IMI+20μmol/L TMX
Inhibition rate	65.51%	67.41%	69.21%	70.21%
Variation	0.00%	1.90%	3.70%	4.70%

### Analysis of actual sample recovery

The detection was carried out using the actual sample recovery test technique, with three sets of experiments carried out in parallel. The results are shown in Table 3. The spiked recoveries of mango, cowpea, and water samples ranged from 98.32% to 102.25%, 99.86% to 107.52%, and 100.24% to 109.96%, respectively, relative standard deviations less than 5%. Therefore, the prepared molecularly imprinted sensors can meet the requirements for the rapid detection of imidacloprid in mango and cowpea samples and water samples.

**Table 3:** Actual sample recovery results.

Samples	Added (μmol/L)	Found (μmol/L)	Recovery (n = 3)	RSD (n = 3)
Mango	1	0.98	98.32%	3.34%
	2	1.98	99.16%	1.58%
	3	3.07	102.25%	4.57%
Water	1	1.08	107.52%	4.45%
	2	2.07	103.51%	1.62%
	3	3.00	99.86%	3.20%
Cowpea	1	1.10	109.96%	3.13%
	2	2.00	100.24%	4.61%
	3	3.09	103.31%	1.38%

## Discussion

Pesticide residue detection methods are currently developing rapidly. More and more immunoassays have been used for the rapid detection of imidacloprid in the last two decades, such as enzyme-linked immunosorbent assay (ELISA) [48], chemiluminescent enzyme-linked immunosorbent assay (CL-ELISA) [49], and time-resolved fluorescence immunoassay (TRFIA) [50], as well as novel methods such as UCNPs immunoassay methods[51].

Electrochemical sensor analysis has good accuracy and sensitivity. The electrochemical sensor based on a screen-printed electrode is more portable and has a lower cost. Owing to the widespread usage of electrochemical technologies, researchers at home and abroad have developed a range of electrochemical detection methods for in recent years. *Ghodsi et al*. [52] developed an electrochemical sensor for imidacloprid based on electrodeposited TiO_2_ nanoparticles (TiO_2_ NPs) modified glassy carbon electrode (GCE) with a detection limit of 0.3 μM.

Majidi *et al*. [53] introduced copper phthalocyanine (CuPc) into the carbon-ceramic network. A new sol-gel electrocatalytic carbon ceramic electrode was prepared. The imidacloprid and the imidacloprid residues in the commercial preparations were determined. The results obtained from the commercial formula were identical to those obtained by standard high-performance liquid chromatography (HPLC).

Chenggen Xie *et al*. [54] used molecular imprinting technique to synthesize polymers with chlorpyrifos imprint and used chemiluminescence to determine with a detection limit of 0.92 μM. Electrochemical detection using the molecular imprinting method has also been studied at home and abroad. Dai [55] used bensulfuron and imidacloprid as template molecules and polysulfide cordial as electrochemical probes to construct a dual-template dual signal imprinted electrochemical sensor for the sequential detection of two template molecules. NourddineA *et al*. [56] catalyzed the reduction of imidacloprid using a metallic silver electrode (MSE) in Britton-Robinson (B-R buffer, pH = 11.6). Zhao *et al*. [57] fabricated a reduced graphene oxide/cyclodextrin modified glassy carbon electrode (rGO/CD/GCE) to detect imidacloprid.Zhai *et al*. [58] prepared a simple sensor based on oligosaccharide-modified three-dimensional graphene (OCS-3D-G) stand-alone electrode for the electrochemical determination of the insecticide imidacloprid. *Guo et al*. [47] combined the benefits of CS with the structural characteristics of F-CNTs to create a unique imprinted electrochemical sensor with selective detection of imidacloprid utilizing both materials, which employed chitosan as a functional substrate, but the modifiers used were different.

The electrochemical detection based on molecular imprinting has been studied for imidacloprid residues. In current study, we used a handmade portable sensor to analyze the samples more quickly and efficiently. A molecularly imprinted sensor with excellent performance was prepared by modifying the screen-printed electrode (SPCE) with the home-made sensor using the cell phone software of the electrochemical detection system designed by ourselves. Its performance tests showed good repeatability and interference resistance, and the spiked recovery test results for real samples were good. Comparing the results of the electrochemical workstation with those of the portable sensor used in this experiment, the portable sensor used in this study has a better linear fit for the inhibition rate detection, and therefore has a better performance compared with the electrochemical workstation. However, the scanning curve fluctuated slightly. Its scan stability was still not as good as that of the electrochemical workstation, indicating that additional study is needed to improve the detection device and electrode fabrication. In addition, imidacloprid is commonly utilized in various crops, including rice, cotton, and wheat. Therefore, we may continue to increase the sensor’s detection range or use it to detect other types of pesticide residues in the future, ensuring the safety of agricultural products and the environment.

## Conclusion

**Fig 3:**
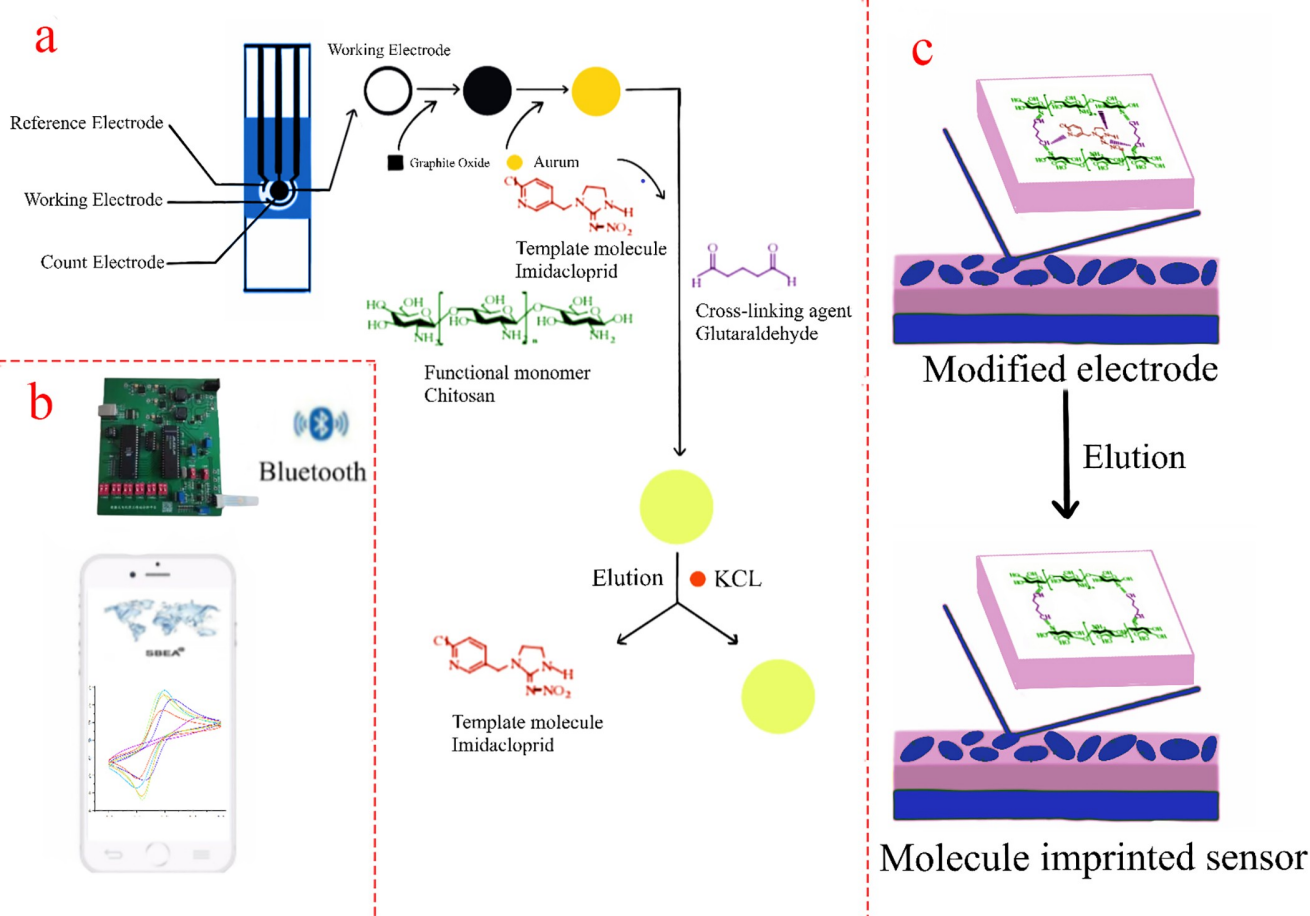
Schematic diagram of molecular imprinted sensor preparation. (a) The modification process of the electrode. (b) Image of detection system based on mobile phone, including micro electrochemical workstation and smart phone connected by Bluetooth. (c) Internal structural changes of the elution step.

In this study, we prepared imidacloprid as a portable molecularly imprinted sensor with good repeatability and anti-interference by electro reducing graphene oxide on the screen-printed electrode, modifying gold nanoparticles, using chitosan as functional matrix, imidacloprid as template molecule, and glutaraldehyde as cross-linking agent (shown in Fig 3). The peak response current of the sensor showed a good linear relationship with the concentration of imidacloprid. The recoveries of imidacloprid in mango, cowpea, and water samples were 90–110% (RSD < 5%), which proved the feasibility and practicability of the sensor, It can lay a foundation for the detection of imidacloprid pesticide residues in food or environment.The developed method does not need complex pretreatment process, and the instrument is light and portable. The pesticide residue detection system can be connected to the Internet, which is convenient for government departments to carry out digital and information management and decision-making.
